# Characterization of the biofilm matrix composition of psychrotrophic, meat spoilage pseudomonads

**DOI:** 10.1038/s41598-020-73612-0

**Published:** 2020-10-05

**Authors:** Nirmani N. Wickramasinghe, Mya M. Hlaing, Joshua T. Ravensdale, Ranil Coorey, P. Scott Chandry, Gary A. Dykes

**Affiliations:** 1 School of Public Health, Curtin University, Bentley, WA 6102 Australia; 2 CSIRO, Agriculture and Food, Werribee, VIC 3030 Australia; 3 School of Molecular and Life Sciences, Curtin University, Bentley, WA 6102 Australia; 4 Graduate Research School, Curtin University, Bentley, WA 6102 Australia

**Keywords:** Microbiology, Applied microbiology, Biofilms

## Abstract

Psychrotrophic *Pseudomonas* species are the key spoilage bacteria of aerobically stored chilled meat. These organisms readily form biofilms on meat under refrigerated conditions leading to consumer rejection and associated economic losses. Limited information is available on the matrix composition of the biofilms formed by these bacteria. We quantified and characterized the main components of the matrix of mono-species biofilms of selected *Pseudomonas fragi* and *Pseudomonas lundensis* strains using chemical analysis and Raman spectroscopy. The biofilms were grown at 10 °C and 25 °C on nitro-cellulose membranes placed on surface sterilized beef cuts. Extra-cellular polymeric substances of the matrix were extracted in soluble and bound forms and were chemically assessed for total carbohydrates, proteins and extra-cellular DNA. Both *Pseudomonas* species showed a significant increase in total carbohydrates and total proteins when grown at 10 °C as compared to 25 °C. Extra-cellular DNA did not show a strong correlation with growth temperature. Raman spectra were obtained from planktonic bacteria and membrane grown biofilms at 10 °C and 25 °C. Higher levels of guanine were detected in planktonic cells as compared to biofilm cells. This study suggests that psychrotrophic *Pseudomonas* species may respond to cold stress by increasing extra-cellular polymer secretions.

## Introduction

Meat is a rich source of nutrients with high water activity which makes it a highly perishable food commodity^1^. Psychrotrophic pseudomonads are the main cause of organoleptic degradation of aerobically stored chilled meat^2^. These organisms are metabolically diverse and can withstand the stressful environmental conditions of chilled storage as well as competition from other psychrotrophic organisms on meat^3,4^. A key characteristic of psychrotrophic pseudomonads is that they readily form biofilms under chilled storage^5^. When these biofilms combine with meat exudates it leads to slime formation which is an important quality defect which leads to consumer rejection of meat.

Although considerable research has been undertaken on planktonic spoilage pseudomonads in broth culture models, limited information is available about their biofilm formation as well as how biofilms contribute to their predominance on meat. Biofilms are formed when bacterial cells attach themselves irreversibly to a surface or to each other and embed in a self-produced and/or an acquired exo-polymeric matrix^6,7^. These sessile groups of bacteria exhibit different phenotypic characteristics as compared to their planktonic counterparts^8^.

The exo-polymeric matrix of biofilms protects the bacteria against harmful environmental conditions such desiccation, radiation, predation and antimicrobial compounds^9,10^. The matrix immobilizes microorganism and aids in quorum sensing, horizontal gene transfer and enzymatic reactions^11^. It also aids in cellular arrangement, and provides mechanical stability which affects the overall structural arrangement of the biofilm^6,11^. The biofilm matrix is therefore more than an inert material and warrants study in detail. In order to minimize and control biofilm formation, a thorough understanding of the matrix components and their proportions is essential. Biofilm matrix is typically composed of water, extra-cellular polymeric substances (EPS), extra-cellular DNA, lipids and extra-cellular vesicles^12,13^. EPS are high molecular weight substances and are often be divided into two main fractions known as soluble EPS (SEPS) and bound EPS (BEPS)^14^**.**

*Pseudomonas fragi* and *Pseudomonas lundensis* are two important species that cause spoilage of chilled meat globally^3^. Since biofilms provide many benefits to protect the residing bacteria from harmful environmental conditions, it is likely that the ability to form biofilms is advantageous to psychrotrophic *Pseudomonas* spp. To date, limited information is available on the mechanisms of biofilm formation by these organisms and its associated structure and matrix composition.

Since the environmental conditions are known to affect the metabolism of biofilms, it is important to find out if there are differences in the matrix composition between biofilms formed at chilled and ambient temperature conditions. A key objective of this research was to assess if psychrotrophic *P. fragi* and *P. lundensis* respond to low temperature by changing their matrix composition or quantity. For this reason 10 °C was selected to mimic low but temperature abuse conditions that can result during handling of chilledmeat^15,16^ . The ambient temperature which has the best optimal growth for these two species is reported to be 25 °C^17,18^.

This study characterizes the key extra-cellular polymeric compounds of biofilm matrix of *Pseudomonas fragi* and *Pseudomonas lundensis* strains when grown under lower (10 °C) and ambient temperature (25 °C) conditions using chemical and spectroscopic methods. It further investigates differences in the chemical composition between bacterial modes of growth by comparing the Raman spectral profiles of planktonic and biofilm cultures of these species.

## Results

### Cell counts in biofilms

For biofilm matrix studies to be comparable between the two different temperatures, biofilm components were extracted at approximately similar levels of maturity. As bacterial growth rate within the biofilm was dependent on temperature, the level of maturity was determined by the number of cells in *P. fragi* and *P. lundensis* biofilms formed on nitro-cellulose membranes at 10 °C and 25 °C over 7 days. Based on the data, day 5 was selected for biofilms grown at 25 °C and day 6.5 for biofilms grown at 10 °C. At these time points the numbers of bacteria in the biofilms for each of the strains are similar as presented in Table 1.

**Table 1:** Cell counts in mono-species biofilms of the *P. fragi* and *P. lundensis* strains grown at 25 °C and 10 °C on nitro-cellulose membranes placed on meat.

Bacterial strain	Log CFU/cm^2^ at 25 °C on day 5	Log CFU/cm^2^ at 10 °C on day 6.5
*P. fragi* 1793	11.5	11.2
*P. fragi* 1832	11.3	11.5
*P. lundensis* 1822	12.1	11.4
*P. lundensis* ATCC 49968	11.9	10.6

### Matrix protein content

**Figure 1:**
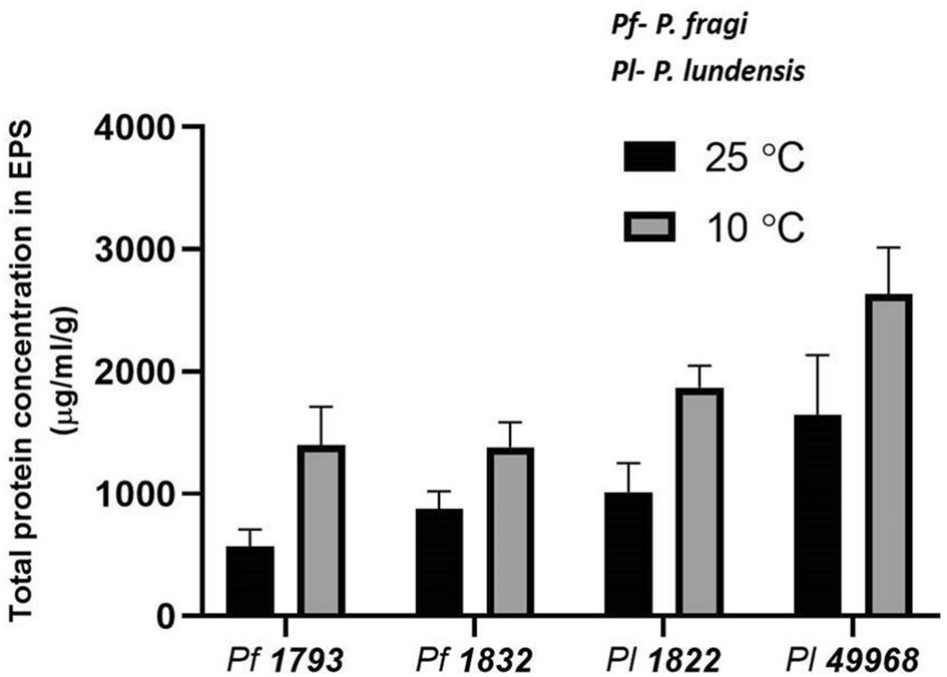
The total protein content of the matrix EPS. The protein content of soluble and bound fractions of extracted EPS of *P. fragi* (1793 and 1832) and *P. lundensis* (1822 and ATCC 49968) biofilms formed on nitro-cellulose membrane placed on meat at 10 °C and 25 °C.

**Figure 2:**
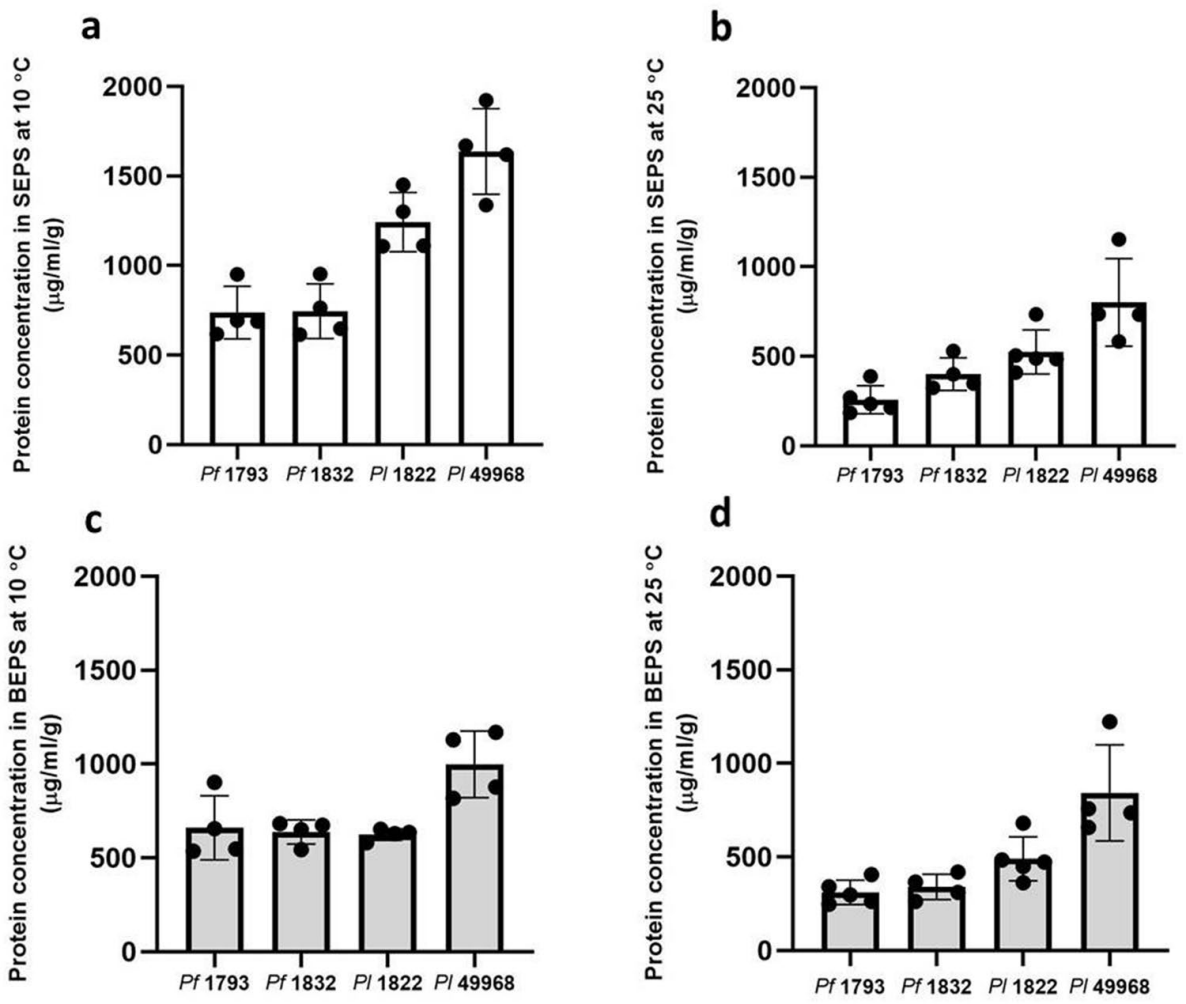
The protein content of the soluble and bound fractions of matrix EPS. The protein content of four biological replicates of *P. fragi* (1793, 1832) and *P. lundensis* (1822, ATCC 49968) biofilms formed on nitro-cellulose membrane placed on meat at 10 °C (**a**,**c**) and 25 °C (**b**,**d**). Error bars show the standard deviations from four biological replicates. Statistical differences were evaluated through one-way ANOVA, with a confidence level of 95% (*P* < 0.05).

At both the growth temperatures, the highest content of total protein was detected in the matrix of the two *P. lundensis* strains as compared to the *P. fragi* strains. When formed at 25 °C the total protein content of the *P. lundensis* ATCC 49968 matrix was 1644 µg/ml/g and when formed at 10 °C it was significantly higher (*P* = 0.019) at 2635 µg/ml/g, a 1.6 fold increase (Fig. 1). When formed at 25 °C the total protein content of the *P. lundensis* 1822 was 1013 µg/ml/g and when formed at 10 °C it was significantly higher (*P* < 0.000) at 1867 µg/ml/g, a 1.84 fold increase (Fig. 1). For *P. lundensis* at the lower temperature there was greater protein content in the soluble EPS than in the bound EPS, while at the higher temperature the levels of protein were similar (Fig. 2). When formed at 25 °C the total protein content of the *P. fragi* 1793 matrix was 568 µg/ml/g and when formed at 10 °C it was significantly higher (*P* = 0.001) at 1397 µg/ml/g, a 2.45 fold increase (Fig. 1). When formed at 25 °C the total protein content of the *P. fragi* 1832 matrix was 877 µg/ml/g and when formed at 10 °C it was significantly higher (*P* = 0.001) at 1382 µg/ml/g, a 1.57 fold increase (Fig. 1). By comparison to the *P. lundensis* strains, the *P. fragi* strains produced a lower quantity of proteins in both soluble and bound EPS fractions. However, the matrix of both *P. fragi* strains had a relatively higher total protein under low temperature in both soluble as well as bound EPS than at ambient temperature (Fig. 2).

### Matrix carbohydrate content

**Figure 3:**
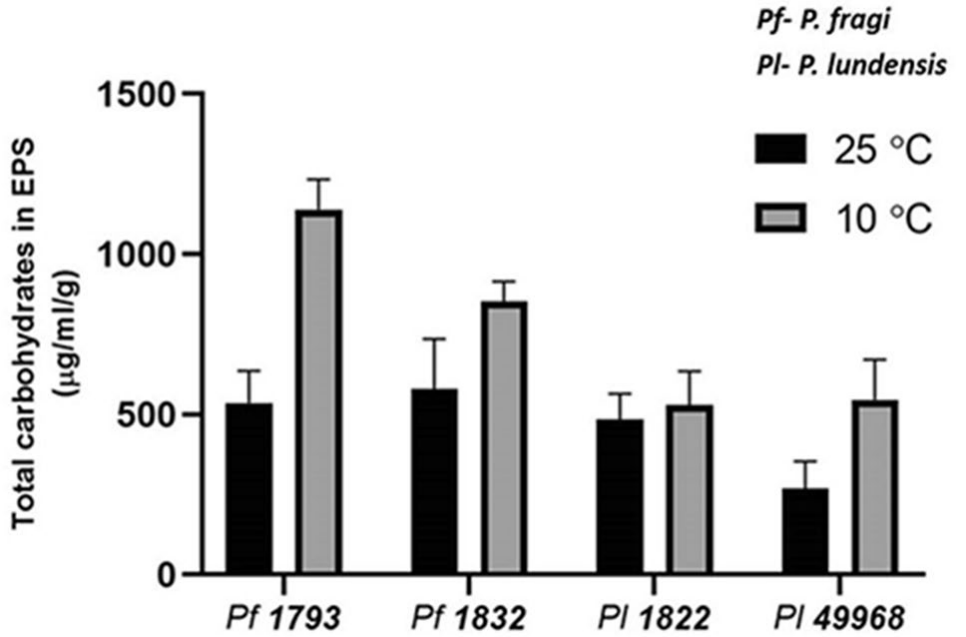
The total carbohydrate content of the matrix EPS. The carbohydrate content of soluble and bound fractions of extracted EPS of *P. fragi* (1793 and 1832) and *P. lundensis* (1822 and ATCC 49968) biofilms formed on nitro-cellulose membrane placed on meat at 10 °C and 25 °C.

**Figure 4:**
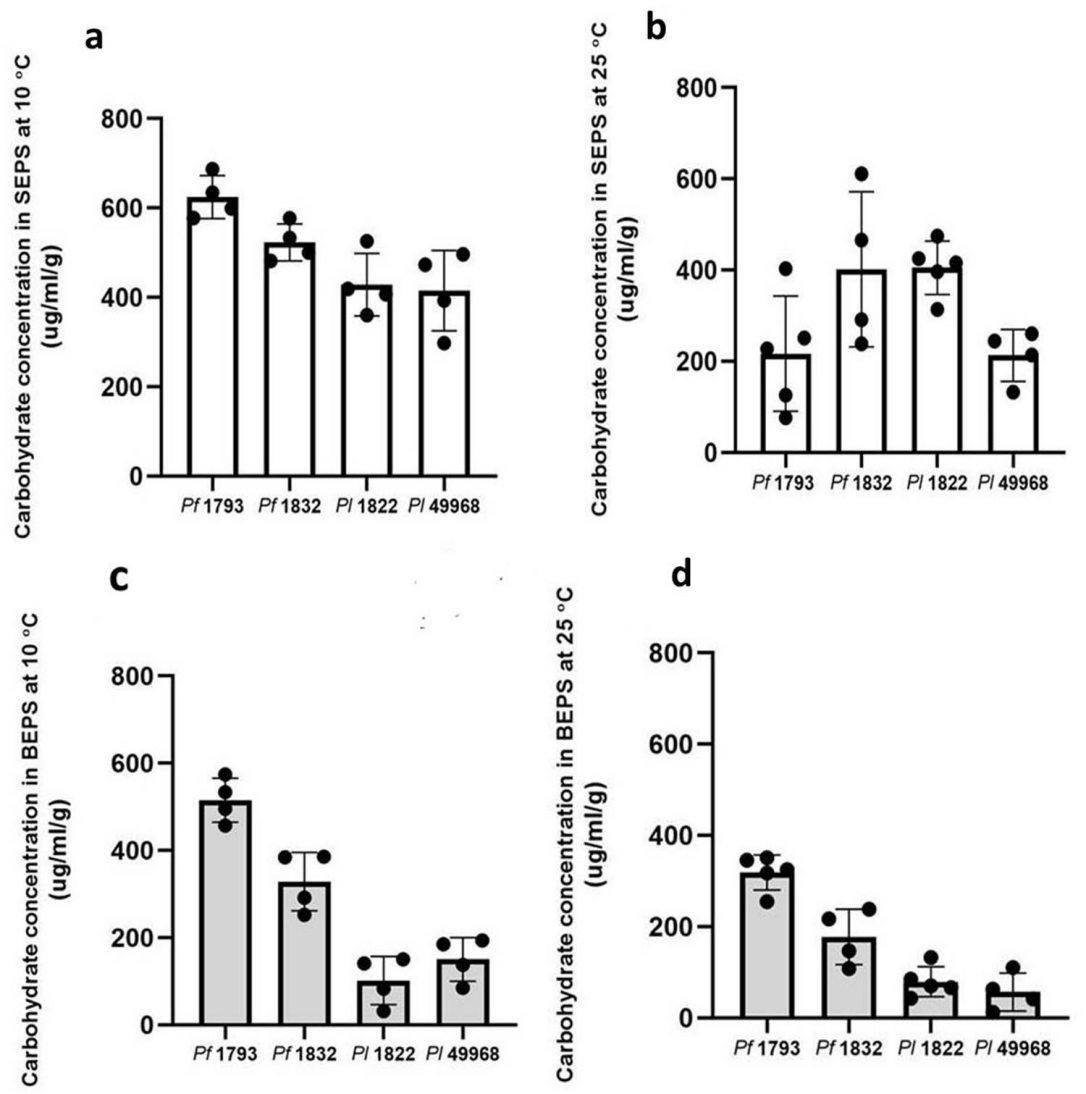
The carbohydrate content of the soluble and bound fractions of matrix EPS. The carbohydrate content of four biological replicates of *P. fragi* (1793, 1832) and *P. lundensis* (1822, ATCC 49968) biofilms formed on nitro-cellulose membrane placed on meat at 10 °C (**a**,**c**) and 25 °C (**b**,**d**). Error bars show the standard deviations from four biological replicates. Statistical differences were evaluated through one-way ANOVA, with a confidence level of 95% (*P* < 0.05).

At both the growth temperatures, the highest content of total carbohydrate was detected in the matrix of the two *P. fagi* strains as compared to the *P. lundensis* strains. When formed at 25 °C the total carbohydrate content of the *P. fragi* 1793 matrix was 535 µg/ml/g and when formed at 10 °C it was significantly higher (*P* = 0.000) at 1140 µg/ml/g, a 2.1 fold increase (Fig. 3). When formed at 25 °C the total carbohydrate content of the *P. fragi* 1832 matrix was 579 µg/ml/g and when formed at 10 °C it was significantly higher (*P* = 0.018) at 851 µg/ml/g, a 1.5 fold increase (Fig. 3). When formed at 25 °C the total carbohydrate content of the *P. lundensis* ATCC 49968 matrix was 245 µg/ml/g and when formed at 10 °C it was significantly higher (*P* = 0.011) at 511 µg/ml/g, a 2.1 fold increase (Fig. 3). The total carbohydrate content of the *P. lundensis* 1822 matrix produced at different temperatures were not significantly different (*P* = 0.485) from each other. Bacterial strains of both species had higher levels of total carbohydrates in the extracted matrix EPS when biofilms formed under low temperature as compared to ambient temperature. Furthermore, the *P. lundensis* strains had higher amounts of carbohydrates in the soluble fraction of the matrix EPS compared to the bound fraction (Fig. 4).

The total protein to total carbohydrate ratio was higher in *P. fragi* strains 1793, 1832 and in *P. lundensis* 1822 when formed under low temperature conditions compared to 25 °C (Table 2). Also, for *P. lundensis* strain ATCC 49968, the significance of increase in matrix protein content (*P* = 0.011) was higher than the increase in matrix carbohydrate content (*P* = 0.019) at low temperature.

**Table 2:** Total protein to carbohydrate ratios of the biofilm matrix of the *P. fragi* and *P. lundensis* strains grown at 10 °C and 25 °C.

Temperature (°C)	*P. fragi* 1793	*P. fragi* 1832	*P. lundensis* 1822	*P. lundensis* ATCC 49968
10	1.2	1.6	3.5	4.7
25	1.1	1.3	2.1	6.1

### Matrix extra-cellular DNA (eDNA) content

**Figure 5:**
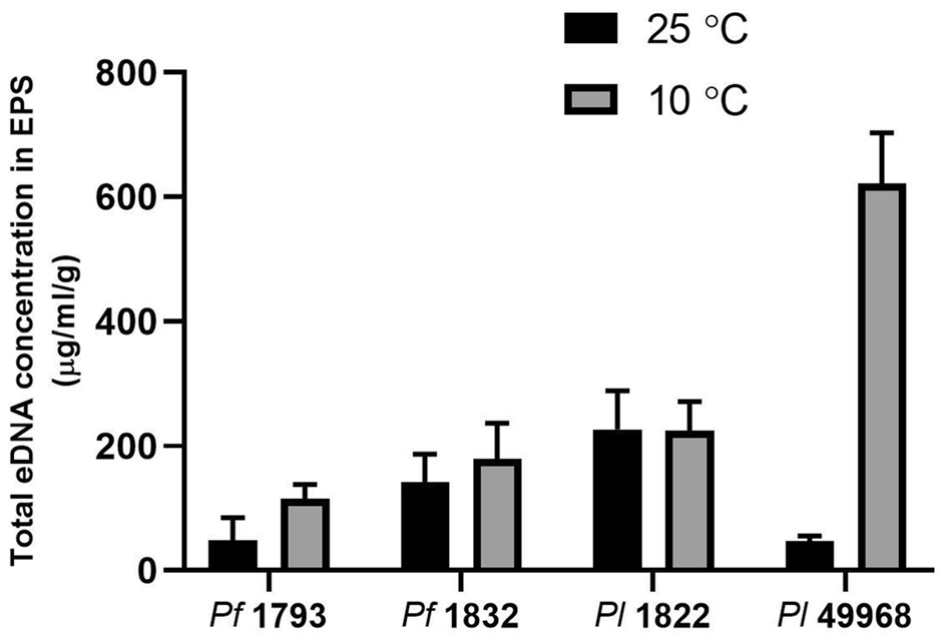
The eDNA content of the matrix EPS. The eDNA content of soluble and bound fractions of extracted EPS of *P. fragi* (1793 and 1832) and *P. lundensis* (1822 and ATCC 49968) biofilms formed on nitro-cellulose membrane placed on meat at 10 °C and 25 °C.

**Figure 6:**
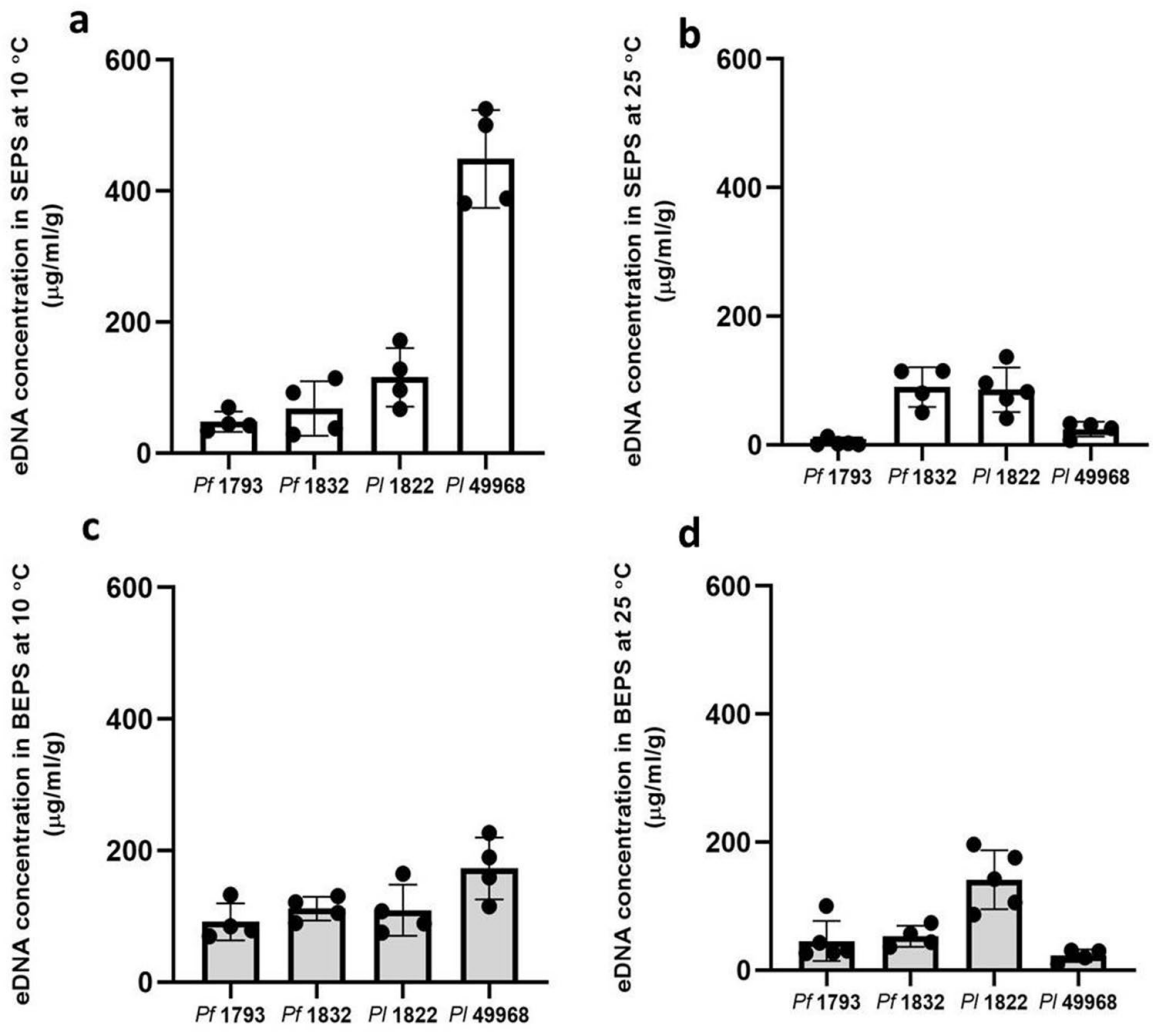
The eDNA content of the soluble and bound fractions of matrix EPS. The eDNA content of four biological replicates of *P. fragi* (1793, 1832) and *P. lundensis* (1822, ATCC 49968) biofilms formed on nitro-cellulose membrane placed on meat at 10 °C (**a**,**c**) and 25 °C (**b**,**d**). Error bars show the standard deviations from four biological replicates. Statistical differences were evaluated through one-way ANOVA, with a confidence level of 95% (*P* < 0.05).

A clear association between the matrix eDNA content and bacterial species, or between matrix eDNA content and temperature level was not apparent in this study. When formed at 25 °C the matrix eDNA content of the *P. lundensis* ATCC 49968 matrix was 47 µg/ml/g and when formed at 10 °C it was significantly higher (*P* < 0.000) at 622 µg/ml/g, a 13.2 fold increase (Fig. 5). Interestingly, this strain had the lowest total eDNA content in the matrix out of the four strains when grown at ambient temperature. When formed at 25 °C the matrix eDNA content of the *P. fragi* 1793 matrix was 51 µg/ml/g and when formed at 10 °C it was significantly higher (*P* = 0.004) at 142 µg/ml/g, a 2.9 fold increase (Fig. 5). Difference in eDNA content could not be detected at the different temperatures for the *P. fragi* 1832 matrix (*P* = 0.342) or the *P. lundensis* 1822 matrix (*P* = 0.954). The eDNA content in both SEPS and BEPS increased by several folds across the biological replicates (Fig. 6).

### Raman spectroscopy

**Figure 7:**
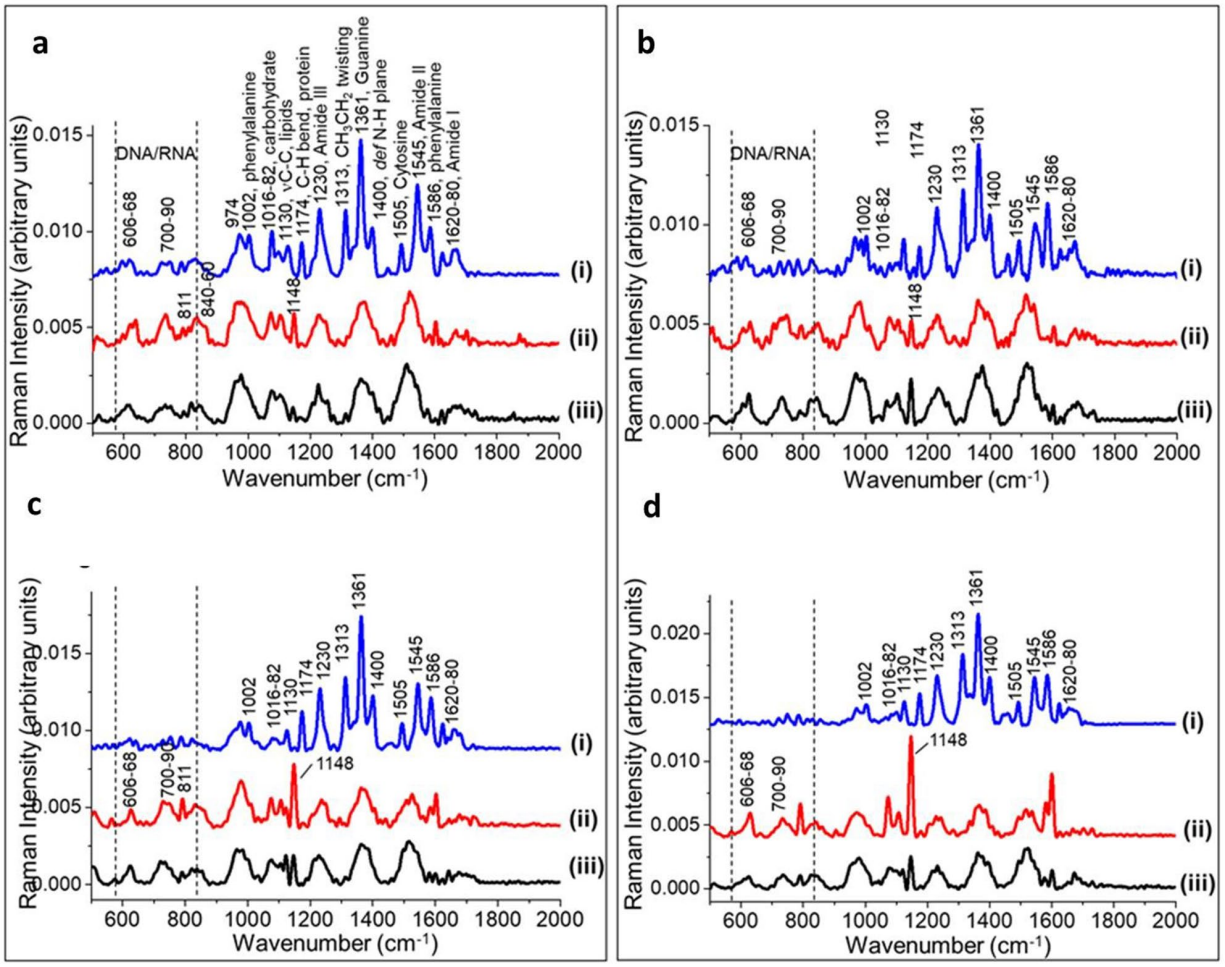
Averaged, intensity-normalised, background subtracted Raman spectra from planktonic cells (i) and biofilms grown at 10 °C (ii) and 25 °C (iii) of four bacterial strains [*P. fragi* 1793 (**a**), *P. lundensis* 1822 (**b**), *P. fragi* 1832 (**c**), *P. lundensis* 49968 (**d**)]. The dominant peaks for DNA/RNA, proteins and carbohydrate are shown with the peak assignments listed in Table 3.

Averaged, intensity-normalised Raman spectra of planktonic bacteria grown at 25 °C and biofilms formed on nitro-cellulose membranes placed on meat at 10 °C and 25 °C were assessed for differences in spectral intensity. Strain level differences can be seen in the intensities of planktonic and biofilm Raman spectra (Fig. 7). The spectra of the four different strains showed many similar peaks that can be assigned to cellular constituents associated with DNA/RNA, proteins, lipids, carbohydrates, based on previous studies as summarized in Table 3. Since Raman spectroscopy analysis is a phenotypic method, all related environmental factors (i.e. growth media and conditions) as well as physiological states of cells can influence in identifying the Raman peaks between different species, or even within the same species or strains^19^. Moreover, it has been reported that certain bacterial species produce biocomponents which may contribute as specific Raman-spectral fingerprints to them^20^.

**Table 3:** Selected Raman frequencies and their peak assignments for the spectra shown in Fig. 7.

Wave number (cm^−1^)	Peak assignment
**DNA/RNA**	
668 (606–68)	T, G (ring breathing)
726, 746	A, T (ring breathing mode of DNA/RNA bases)
781	U, T, C (ring breathing modes in the DNA/RNA bases)
785	U, T, C (ring breathing), PO_2_^−^ *str*. of DNA backbone
811	PO_2_^−^ str. RNA
621–46	C–C twisting mode of phenylalanine (proteins)
974	Ribose vibration, one of the distinct RNA modes
1070–90	Symmetric PO_2_^−^ *str* of DNA (represents more DNA in cell)
**Protein/lipid/carbohydrate/polysaccharide**	
840–60	Polysaccharide structure
1002	Phenylalanine (ring breathing, symmetric)
1105, 1149	Carbohydrates peak for solutions, solids
1171–74	Tyrosine, phenylalanine, C–H bend (protein)
1230–300	Amide III (arising from coupling of C–N stretching and N–H bonding)
1313	CH_3_CH_2_ twisting mode of collagen/lipid
1325–30	CH_3_CH_2_ wagging mode in purine bases of nucleic acids
1361–5	Guanine
1400	NH in-plane deformation
1460	CH_2_/CH_3_ deformation of lipids and collagen
1506–15	Cytosine
1544–5	Amide II
1586–8	Phenylalanine, hydroxyproline
1622	Tryptophan
1655–80	T, G, C (ring breathing modes of the DNA/RNA bases), amide I (protein)

*A* adenine, *C* cytosine, *G* guanine, *PO*_2_^−^ phosphate, *str* stretching, *T* thymine, *U* uracil. Assignments are based on studies in the references.

The Raman spectra of planktonic samples had more prominent peaks than the Raman spectra of biofilm samples grown under both temperatures (Fig. 7). Prominent peaks of biofilm and planktonic samples are listed in Table 3. The sharp peaks in the planktonic spectra can be assigned to proteins (Amide III and II bands found at 1230 cm^−1^ and 1545 cm^−1^) whereas the CH_3_CH_2_ twisting mode of collagen/lipid can be found at 1313 cm^−1^ (Table 3). The bands at 1400 cm^−1^ and at 1171–4 cm^−1^ were related to N–H plane deformation and tyrosine phenyl alanine CH bend, respectively (Fig. 7), (Table 3). The most prominent peak found in the planktonic Raman spectra of all the strains was guanine (1361–5 cm^−1^).

The spectral peaks of biofilm samples were comparatively less sharp. The key peaks which were particularly prominent in biofilm samples were 606–668 cm^−1^ assigned to T, G (ring breathing), 700–90 cm^−1^ assigned to A, T, U, C (ring breathing modes in the DNA/RNA bases), 811 cm^−1^ assigned to PO_2_^−^ str. RNA and 974 cm^−1^ assigned to ribose vibration, which is one of the distinct RNA modes. Compared to other biofilm samples, pronounced differences can be seen in the spectral peaks of biofilms of *P. fragi* 1832 and biofilms of both *P. lundensis* strains grown at 10 °C. High levels of carbohydrates and phenylalanine were detected at 1148 cm^−1^ and 1595 cm^−1^ respectively (Fig. 7).

### PCA analysis of Raman spectra

**Figure 8:**
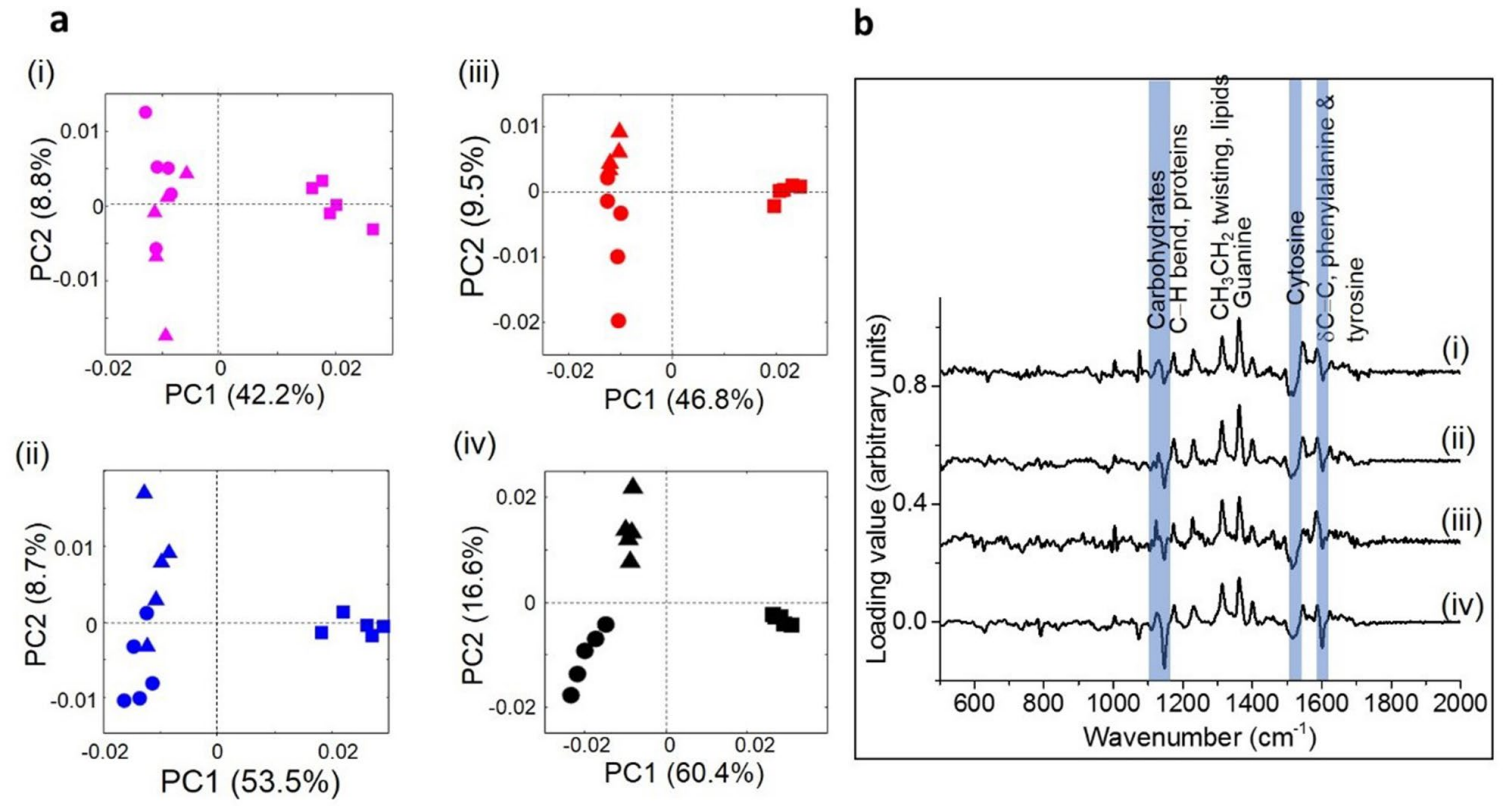
(**a**) Scatter plots of principal component analysis of the Raman spectra from planktonic cells and biofilms of the four bacterial strains: (i) *P. fragi* 1793, (ii) *P. fragi 1832*, (iii) *P. lundensis* 1822 and (iv) *P. lundensis ATCC* 49968. (square, planktonic cells; circle, biofilms grown at 10 °C; triangle, biofilms grown at 25 °C). (**b**) The loading plots. The corresponding PC1 loading plots of each strain exhibit the spectral differences of each comparison: (i) *P. fragi* 1793, (ii) *P. fragi* 1832, (iii) *P. lundensis* 1822 and (iv) *P. lundensis* ATCC 49968.

Principal component analysis was carried out for averaged, intensity normalised Raman spectra of planktonic cells and biofilms of all four strains. Scatter plots of the first and second principal components (PC1 and PC2) show a significant separation between planktonic cells and biofilm samples for each bacterial strain (Fig. 8a).

The first principal component (PC1) accounted for about 45% and 49% of the variance between planktonic and biofilm samples for *P. fragi* strains 1793 and 1832, respectively (Fig. 8a). The PC1 for *P. lundensis* accounted for 61% and 58% of the variance between planktonic and biofilm samples of strains 1822 and ATCC 49968, respectively (Fig. 8a). The chemical changes responsible for the variations between planktonic and biofilm spectra can be observed in the PCA loadings plots (Fig. 8b).

The first principal component loading plot (Fig. 8b) shows peaks at 1180 cm^−1^, 1600 cm^−1^ (associated with proteins), 1310 cm^−1^ (associated with carbohydrates), 1500 cm^−1^ (associated with cytosine) and 1600 (associated with phenylalanine and tyrosine). The peaks which could be used as the chemical fingerprint regions to differentiate biofilm and planktonic samples are associated with carbohydrates, proteins (phenylalanine and tyrosine), DNA/RNA synthesis (cytosine) and lipids. The loading plot has a similar trend for all four bacterial strains.

**Figure 9:**
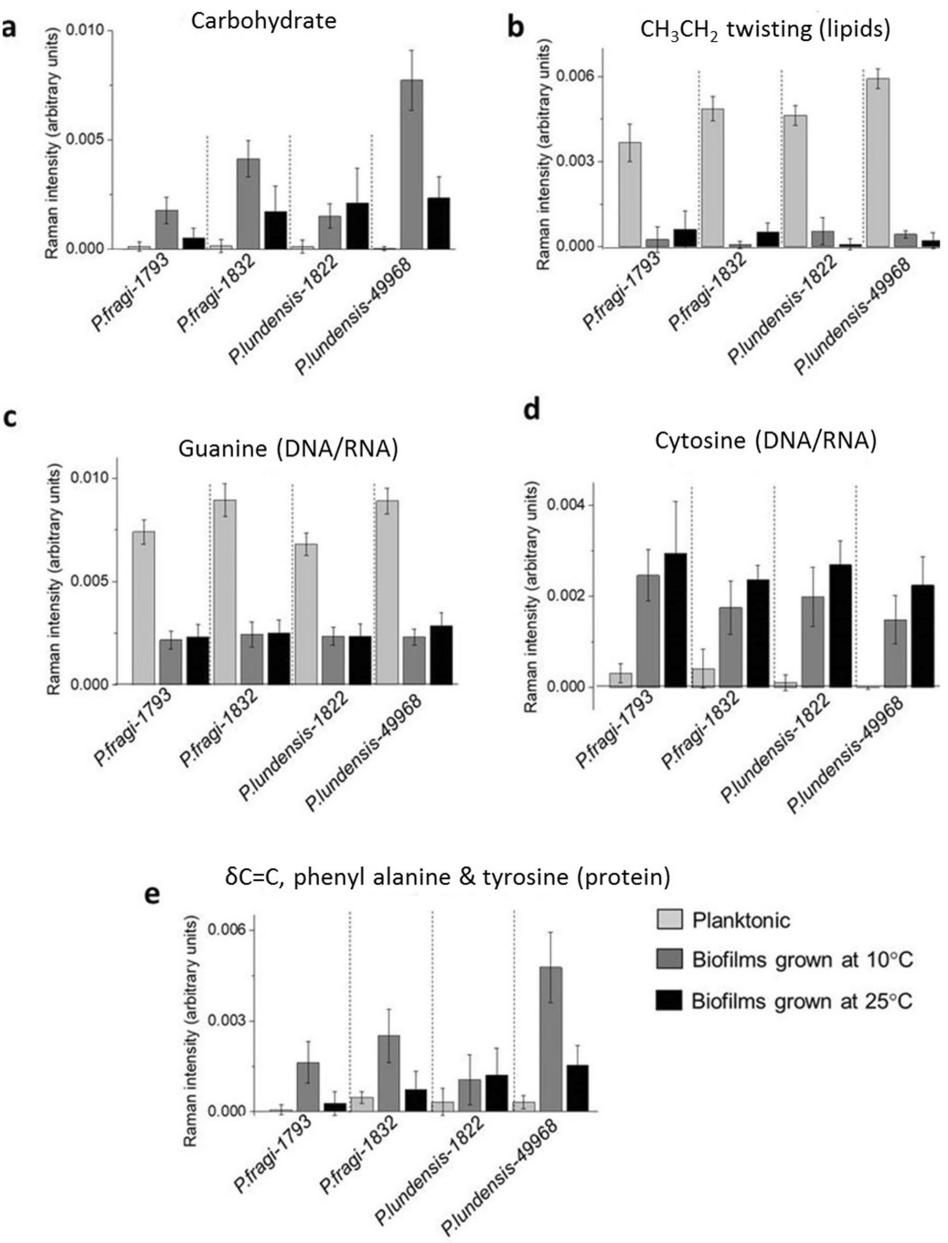
Specific peak analysis of the Raman spectra of planktonic and biofilms of the four bacterial strains. Univariate analysis was performed on the normalised intensity of carbohydrate (**a**), lipid (**b**), DNA/RNA (**c**,**d**) and protein (**e**) structure-specific peaks in the Raman spectra taken from planktonic and biofilm samples.

The specific peaks were selected from the loading plots and the average and the standard deviations of biological replicates of Raman spectra were calculated (Fig. 9). Specific peak analysis from the loading plots showed that the carbohydrate concentration was higher in biofilm samples of all four strains compared planktonic samples. Also,the carbohydrate content was higher in all biofilms formed at 10 °C compared to biofilms formed at 25 °C (Fig. 9a). The lipid content was higher in planktonic bacteria of *P. fragi* 1832 and in both *P. lundensis* strains. However, for *P. fragi* 1793, the lipid content was higher in biofilms grown at 25 °C as compared to planktonic bacteria (Fig. 9b). Guanine was detected at levels several folds higher in all planktonic samples compared to biofilm samples formed at both temperature levels (Fig. 9c). Cytosine was present in several folds higher levels in biofilms samples compared to planktonic samples of all the strains (Fig. 9d). Cytosine levels in biofilms formed at 25 °C were significantly higher (*P* < 0.05) in *P. fragi* 1832 and in *P. lundensis* strains when grown at 10 °C. According to the results, the protein concentration, as indicated by phenylalanine and tyrosine, was high in biofilms of *P. fragi,* 1793 and 1832, and in *P. lundensis* ATCC 49968 grown at 10 °C (Fig. 9e).

## Discussion

Biofilms are a dominant form of microbial life and are difficult to be completely eradicated^21^. The biofilm mode of life provides many advantages to the residing bacteria. The majority of past research studying biofilms of spoilage pseudomonads used broth culture models or abiotic surfaces^22–24^. Biofilms are formed in environments that are vastly different to one another and it is important to characterize them in-situ. The metabolic activities and by-products of microorganisms vary greatly based on the environmental conditions that they are grown upon^25^. The production of EPS is affected by the carbon source^26^, growth conditions and environmental conditions^27^. The model system used in this study closely resembles spoilage conditions on meat muscle under industry applicable conditions.

This study assessed the key matrix constituents and quantity of *P. fragi* and *P. lundensis* biofilms formed under chilled and ambient temperature conditions to assess their response to low temperature. The experimental model used in this study allowed the biofilms to be formed on porous nitro-cellulose membranes placed on surface sterilized meat. The pore size of the membranes allowed adequate access of nutrients and water from meat but prevented the substances of meat being collected during extraction.

According to the CFU calculations, biofilms formed under 10 °C and 25 °C reached an approximately similar CFU counts on days 5 and 6.5. Previous studies characterizing the biofilm matrix of *P. fragi* grown directly on chicken breast pieces under modified atmospheric conditions reported substantially higher levels of protein in the matrix compared to other studies^28–30^. It was concluded that this could be due to proteins collected from the meat interfering with the matrix composition.

This model may somewhat reduce the rate of bacterial growth due to a lack of direct contact with the meat surface. However, by monitoring bacterial cell counts we previously demonstrated that these biofilms follow a similar pattern to biofilms that are formed directly on the meat muscle^5^. Various EPS extraction methods consisting of physical, chemical or a combination of these means are available in literature. It has been found that different extraction methods can yield different results^31,32^. The EDTA extraction method has been shown to be an efficient extraction method with minimal cell lysis^32–34^. The biofilm mechanical breakdown process used in this method prior to chemical extraction via vortex mixing and sonicating increases the efficiency of EPS extraction^35^. Total carbohydrate extracts contain charged and uncharged polysaccharides and EDTA is known to chelate metal ions that form the links between carbohydrate and increase the extraction efficiency^32^. The formamide used in this extraction process enhances the efficiency of bound EPS extraction^36^ and decrease the contamination by intracellular substances^13^.

The EPS were extracted in bound and soluble forms to obtain a better representation of the polymer distribution. According to past research done on EPS, soluble and bound forms are distributed in different layers. Soluble EPS are distributed in outside layers in aqueous phase are weakly adhered to cells and easily dissolved in solutions^37^. Bound EPS closely accumulate on the outside of cells. Tightly bound EPS strongly support the mechanical stability of biofilms^38^. Therefore, changes in SEPS and BEPS quantity can affect changes in the structure of the biofilm**.**

In this study, the key components of matrix EPS of spoilage pseudomonads were assessed. It was demonstrated that the psychrotrophic *Pseudomonas* spp. studied here responded to low temperature conditions by altering their matrix composition. The increase in total carbohydrate content was significantly high when biofilms are formed at low temperature for both *P. fragi* strains and for *P. lundensis* ATCC 49968.The increase in total protein content was statistically significant for all the four strains when the biofilms were grown at 10 °C as compared to 25 °C. Previous studies have found that bio-flocculation of microbes increased with increasing protein content of the matrix^39^. Some studies have also reported an increase in EPS in biofilms due to increased biomass^37^. In our study the increase in EPS secretion under low temperature was not correlated to an increase in the biomass as the biofilms were extracted at approximately similar maturity and cell count levels.

Low temperatures can be considered stressful for microorganisms and stressful environmental conditions are known to increase EPS production^36,40^. The increased EPS production seen in our study is in agreement with past research which quantified the biofilm matrix of *P. lundensis* meat isolates grown in microtiter plates14. In that study, a higher biofilm production was detected by crystal violet staining when biofilms were grown at lower temperatures^24^. This study also reported significant differences between the maximum amounts of biofilm produced at 30, 10 and 4 °C. Similar results were observed for *Pseudomonas putida* which is another psychrotrophic meat spoilage organism. When grown as biofilms on polyvinyl chloride coupons under low and ambient temperatures, and stained with crystal violet, higher levels of biofilms formed at lower temperatures^41^. The EPS and total carbohydrate production of *P. putida* biofilms increased with increasing matrix stress due to water loss^42^. Biofilms formed under high shear stress conditions grow to be more dense and high hydrodynamic shear forces appear to promote production of excessive cellular polysaccharides^43^. Based on the results of our study and past studies it appears that the production of EPS increases under stressful environmental conditions. Apart from providing protection against stressful environmental conditions, EPS also acts as a carbon/energy reservoir during biological process^38^.

An interesting observation of this study is that marked differences can be seen in the levels of carbohydrate and protein between the *P. fragi* and *P. lundensis* biofilm matrix despite the close taxonomic distance between these species^44^. *Pseudomonas fragi* strains had a higher proportion of total carbohydrates and a comparatively lower proportion of total proteins in matrix EPS compared to *P. lundensis*. It has been established that the carbohydrates in the matrix play an important role in a biofilms structure and stability. A previous study by Tay, et al.^45^ established that the disappearance of aerobic sludge granules was closely related to a reduction in cellular polysaccharides and the polysaccharides help to stabilize the biofilm. The high concentration of carbohydrate may therefore help *P. fragi* strains to produce mechanically stable biofilms. Also *P. fragi* had higher carbohydrate content in bound EPS compared to *P. lundensis* strains at 25 °C which indicates the *P. fragi* matrix can be mechanically stronger than *P. lundensis* even at ambient temperature conditions.

Our previous study assessing *P. fragi* and *P. lundensis* biofilm growth directly on sterile beef muscle detected significant differences in microstructural and cellular arrangement between these two species (5). Confocal laser scanning microscopy images of fluorescently stained biofilms showed that *P. fragi* produced highly dense, compact, flat biofilms of vertically oriented cells with limited intercellular gaps. In contrast, *P. lundensis* produced biofilms with loosely arranged cells with considerable intercellular gaps and voids. Studies on *Vibrio cholerae* biofilms which have a similar cellular arrangement to *P. fragi,* biofilms have shown that one of the matrix proteins namely RbmA aids in binding cells closely together^46^. Such matrix material helped to form biofilms with tightly coherent cells which limited the entry of foreign cells into the biofilm and promoted invasion resistance^10,46^. It can be hypothesized that the cellular arrangement of *P. fragi* strains could be a result of the matrix composition. Studies have also found that *P. fragi* becomes the predominant bacterial species on long term stored chilled meat^47^. The cellular arrangement and the matrix composition may aid in their long-term survival.

Extra-cellular DNA is an important component of the biofilm matrix. Extra-cellular DNA is released to the matrix via cellular disruption and/or through membrane vesicles^48^. Compared to other strains, significantly high levels of eDNA and protein levels were detected in *P. lundensis* 49968 when grown at low temperature. It can be hypothesized that this could be due to cellular disruption and cell death of this particular strain when grown at lower temperatures. Significant increases in eDNA levels were not detected in other strains tested when formed at 10 °C. Isolate based variation in metabolic activity of *P. fragi* and *P. lundensis* strains has been reported in previous studies on these strains^49^. Much diversity in volatile organic compound and metabolite production were observed within species when these strains where grown on chilled beef paste^49^. Further studies are necessary with more *P. lundensis* strains to assess if these are genomic DNA released due to cell death or DNA secreted actively.

In this study, there was not a strong correlation between matrix eDNA production and temperature levels. *Pseudomonas fragi* strains 1793 and 1832 and *P. lundensis* ATCC 49968 had a higher eDNA content when grown under low temperature while *P. lundensis* 1822 had a slightly lower eDNA content under low temperature. These results are in agreement with our previous studies, where no significant difference could be detected in the levels of eDNA production between several *P. fragi* and *P. lundensis* strains formed at different temperature levels^5^. Strain level variationin eDNA production is more prominent in *P. fragi* and *P. lundensis* than species level differences.

In this study the total quantities of carbohydrates, proteins and eDNA in the matrix EPS were assessed by chemical analysis. A detailed analysis of the types of carbohydrates and types proteins in the biofilm matrix EPS is important to identify specific functions of these polymers. However, in complex eco-systems such as biofilms, polysaccharides form bonds with proteins and other substances and form complex chemical complexes which make the identification of these compounds difficult. Also, due to the diversity in sugar monomers and linkages, it is difficult to isolate and characterize specific polysaccharides from total carbohydrates in environmental samples^6^. Therefore, chemical analysis should be combined with other analytical methods when studying matrix composition.

An advantage of Raman spectroscopy is that an overall estimation of the chemical components present in a biological substance and their relative abundances can be determined. It is a non-invasive, laser based technology which detects inelastic scattering of the monochromatic light and each chemical vibration is assigned to a specific Raman wavenumber^50^. Staining the matrix components with specific fluorescent dyes can also provide an estimation of the chemical structure in a biofilm^51^. However, such methods have limitations due to the limited availability of specific dyes as well as the limitations in dye penetration^52^. Apart from assessing the total carbohydrates, total proteins and eDNA, the Raman spectra provides a view of the overall chemical composition and its intensities in biofilm and planktonic samples.

This study used Raman spectroscopy to detect biochemical differences between planktonic and biofilm bacteria^53,54^*.* Since media residues were washed off the planktonic bacteria, the Raman spectra obtained from them can be used as a reference to observe the chemical changes that occurred during biofilm formation. Theoretically, the Raman peak intensity is directly proportional to the concentration of the represented chemical constituents^55^.

Based on the Raman spectral intensity it is clear that guanine was present in high levels in planktonic samples as compared to biofilm samples. The exact reason for such a difference is currently unknown. However, a universal trait of all biofilms is the response to secondary messenger cyclic dimeric guanosine monophosphate (cyclic di-GMP)^56,57^. A high cyclic di-GMP level necessary for biofilm formation while a decrease in cyclic di-GMP levels can lead to biofilm dispersal. Guanosine is a nucleoside derived from guanine and ribose. Guanine at biofilm stage could be used for the development of cyclic di-GMP. Thus, the detection of low levels of guanine in biofilms samples could likely be that guanine is utilized for cyclic-di-GMP formation.

According to the results of the loading plots, biofilms contained high levels of phenyl alanine. Some studies have observed an increase in biofilm formation in *Pseudomonas aeruginosa* in the presence of certain amino acids including phenylalanine^58^. According to the Raman spectra, biofilms contain high level of carbohydrates compared to planktonic bacteria. At the same time biofilms grown at 10 °C had high spectral intensity of carbohydrates compared to 25 °C grown biofilms. Past studies have reported that Raman spectra of *Klebsiella pneumonia, Escherichia coli*, and *Pseudomonas aeruginosa* biofilms contained a larger amount of polysaccharides compared to the planktonic cells^59^. However, the same study reported lower spectral peaks for proteins and nucleic acids in biofilms.

The results of Raman spectroscopy correlate with the results of chemical analysis where high carbohydrate and protein contents were detected in low temperature stored samples. The spectral peaks of biofilm samples were less prominent compared to planktonic spectra. This could most likely be due to the presence of large amount of complex matrix material surrounding the cells. Biofilms are highly complex and consist of a heterogeneous mixture of biomolecules that contribute to the collected Raman spectra. Therefore, its spectral pattern can be complex^60^.

The chemical analysis results showed differences in EPS composition between the tested *P. fragi* and *P. lundensis* strains as described above. However, species specific differences in matrix carbohydrate and protein could not be detected using Raman spectroscopy. Also, the differences in the matrix EPS composition between the two temperatures were clearer for all the strains in the chemical analysis data compared to spectroscopic data. These differences are likely caused by the differences in data acquisition. For the chemical analysis, the bacterial cells were separated from the biofilm matrix and EPS were measured from the liquid. For the Raman spectroscopy, the spectra were obtained by focusing on single cells and their immediate surroundings of the membrane grown biofilms^61^. In order to confirm whether the differences in carbohydrate and protein composition are species specific, more bacterial strains need to be assessed. A greater understanding of the composition of the biofilm matrix in situ determined in this study may contribute to establishing mechanisms to disrupt biofilms formation on meat and extend shelf-life.

## Conclusion

Previous studies have tested chemical compounds, including heavy metals such as mercury or copper and proteases, that target and degrade EPS of the biofilm^62,63^. However, heavy metals and large amounts of proteases are not suitable for the control of biofilms formed on meat. Other studies have applied glycosidases, proteases and DNases to and their combinations to degrade the extracellular matrix^64^. The effectiveness of these enzymes can depend on environmental conditions and their effect on meat is unknown. In order to select a suitable approach to target the biofilm matrix on meat, a detailed knowledge of the main matrix components and their proportions under practical industry conditions are important. This study provides an insight into the main matrix components of meat spoilage pseudomonads and their proportions and changes induced during chilled storage. This knowledge is useful for the development of biofilm degrading compounds for food material.

## Materials and methods

### Bacterial culture preparation

Two strains each of *P. fragi* (1793 and 1832) and *P. lundensis* (1822 and ATCC 49968) were selected for this study as being representative of the species based on previous research on biofilm formation by these and other strains^5^. Specifically, *P. fragi* 1793 and *P. lundensis* 1822 had high growth rates^5^. The type strains of each species (*P. fragi* ATCC 4973 (1832) and *P. lundensis* ATCC 49968) were selected as they are points of reference for other strains that may be investigated.

### Preparation of biofilms on meat

Fresh beef ‘eye round’ cuts were purchased from local butchers and transported to the laboratory chilled (3 °C) within 20 min of purchase. The water activity of fresh beef was measured using a Novasina LabSwift-water activity meter and pH of the muscle was measured using a PHM210 Standard pH Meter for each biological replicate to assure consistency.

Meat was surface sterilized by immersing in boiling water for ten minutes. The cooked exterior was aseptically removed inside a sterilized laminar flow hood and the uncooked, raw interior was used for further experiments. The beef was sectioned into slices of 4 mm thickness using a sterilized stainless-steel deli slicer. The slices were sectioned aseptically to fit a 55 cm^2^ petri plate. Nitro-cellulose membranes with an area of 55 cm^2^ and pore size of 0.2 µm (PALL product ID: S80209) were sterilized by keeping each side under ultraviolet light for 15 min. Each beef slice was placed inside a petri dish and a nitro cellulose membrane was placed on the meat and patted gently to stick uniformly to the top surface of the muscle.

Overnight cultures of each bacterial strain were prepared by inoculating a single colony of the selected strains into 5 ml of tryptone soy broth (TSB, Oxoid, Basingstoke, United Kingdom) and incubating for 18–20 h in at 25 °C at 180 rpm in a shaking incubator. Tenfold serial dilutions were made in TSB and 1 ml from the 10^4^ CFU/ml dilution was added to each membrane placed on the beef muscle and spread evenly on the membrane using sterile plastic spreaders. The petri plates were covered with lids and incubated at 10 °C and 25 °C to allow biofilm formation.

### Selection of the extraction time point

In order to select matrix extraction time point at which biofilms were at a similar level of maturity after growth at two different temperatures, total bacterial cell numbers were determined. The counts in biofilms formed on membranes placed on meat were determined from day 1 to day 7. The cell counts were determined by dissolving the matrix in maximum recovery diluent (MRD) (Oxoid, Basingstoke, UK) as described in earlier studies^5^ and plating on *Pseudomonas* isolation agar medium (Oxoid, Basingstoke, United Kingdom).

### Extraction of the matrix

On days 5 and 6.5 (determined to be the extraction time points) the meat slices incubated at 25 °C and 10 °C, respectively, were removed from the incubator. The biofilms which were formed on the membranes were collected gently with a sterile cell scraper and the wet weight of the biofilm was measured. The biofilms were placed separately in 50 ml falcon tubes containing 10 ml of MiliQ water. Then the tubes were vortexed for 1 min at maximum speed and shaken using a mechanical flask shaker (Griffin and Tatlock Ltd, Birmingham, London) at maximum speed for five minutes to disperse the biofilm uniformly in water. The biofilms were then sonicated in an ultrasound water bath (Ultrasonics. Pty. Australia) for five minutes to break the aggregates and then vortexed again for 30 s. Each tube was centrifuged at 2000 g for 15 min at 4 °C using Sigma6-6 k centrifuge. The supernatant was collected as the soluble EPS and filter sterilized using 0.2 µm pore sized membrane filters.

The bottom sediment remaining in the tubes was dissolved in 10 ml of extraction buffer (2 mM Na_2_PO_4._12H_2_O, 4 mM NaH_2_PO_4-._H_2_O, 9 mM NaCl, 1 mM KCl, pH 7) and a 60 µl aliquot of 40% formamide was added. The mixture was incubated at 4 °C in a shaking incubator for 30 min. Subsequently, 10 ml of ethylenediamine tetraacitic acid (EDTA) extraction buffer was added to the biofilm suspension and the mixture was shaken for a further 3.5 h in a shaking incubator at 20 °C at 180 RMP^32^. The flasks were vortex mixed for 30 s and centrifuged at 10,000 g for 20 min. The supernatant was collected as bound EPS and filter sterilized with 0.2 µm pore sized membrane filters^32^. The extracted samples were kept at 3 °C until further analysis. Samples were not frozen to avoid alteration of the chemical composition. Four individual biological replicates were carried out under identical conditions. The soluble and bound EPS were used for chemical quantification of key matrix EPS components. In order to normalize the EPS concentration across samples of different wet weight, the results were divided by the wet weight of the corresponding biofilm. The results are presented as ug/ml per gram of biofilm wet weight.

### Protein content determination

The protein concentrations of the extracted soluble and bound EPS were analyzed using the Qubit protein assay kit (ThermoFisher Scientific-Q33211) according to manufacturer instructions. Three technical replicates were carried out for each sample. Protein concentration was normalized by dividing values by the corresponding wet weight of the samples.

### Carbohydrate content determination

Total carbohydrates in the extracted soluble and bound EPS of each sample were quantified using the total carbohydrate assay kit (Sigma, MAK104-1KT) according manufacturer’s instructions. The assay was prepared in 96 well plates with a series of glucose standards. Three technical replicates were carried out for each standard and sample. The concentration of the total carbohydrates in each sample was calculated using the standard curve. The amount of total carbohydrates in each sample was normalized by dividing values by the wet weight of the corresponding biofilm.

### Extra-cellular DNA concentration determination

The Qubit dsDNA Assay Kit (Molecular Probes, Europe-Q33230) was used to determine the eDNA concentration in soluble and bound EPS samples according to manufacturer instructions. The eDNA concentration in each sample was normalised by dividing values by the corresponding wet weight of the biofilm.

### Planktonic cell Raman spectra acquisition

Overnight cultures of strains were prepared as previously described. The cultures were centrifuged at 10,000 g for 5 min. The supernatant was removed, and the collected precipitates were washed twice with MiliQ water to remove traces of broth media. A 10 ul aliquot of the washed culture was placed on a calcium fluoride (CaF_2_) microscope slide and air dried for 10 min. The dried culture was analysed using a confocal Raman spectroscope (Renishaw inVia, Renishaw plc.,Wotton-under-Edge, UK). Specifically, a Raman spectrometer equipped with a Leica microscope plus a deep depletion charge-coupled device detector, 2400 lines per mm grating and a holographic notch filter with slit size of 65 µm was used. As previously described by Hlaing et al.^61^ the incident laser power was adjusted to 1.5 mW of 514 nm radiation from the Argon laser. An estimated spatial resolution on the order of 0.8 µm was used for acquiring the spectra from each sample. The system was calibrated and monitored using a silicon reference (520.5 cm^-1^) before the measurements. A single bacterial cell was focused for each measurement using a 20 × microscope objective (NA = 0.4 in air). The accumulation time for each acquisition was 10 s and single accumulation was collected for a single measurement over the confocal region containing the selected cell. The same pre-processing steps were carried out for obtaining the Raman spectra for planktonic and biofilm samples.

### Biofilm Raman spectra acquisition

Raman spectra were obtained from biofilms grown on membranes incubated at 25 °C and 10 °C on days 5 and 6.5. The membranes were carefully removed with sterile forceps and gently washed with MilliQ water to remove any planktonic bacteria. The biofilms were then air dried for 30 min. The membranes were placed on a CaF_2_ cube and Raman spectra were obtained as described above.

### Raman data processing

The spectra were collected in the 2000 to 500 cm^−1^ range that covers the fingerprint region of most biological materials^65^. The cosmic peaks in the obtained spectra were removed using WiRE 3.4 Raman software integrated in the Renishaw inVia Raman spectroscopy system. The spectra intensities were normalised using total intensity normalisation to remove sample-to-sample variations and the background was subtracted^66^. Raman spectrum of nitrocellulose membrane provided a consistent background signal at 846 cm^−1^ and 1282 cm^−1^. To recover the spectra from the bacterial cells, the peak intensities of the bacterial spectra collected from an intact biofilm isolated on the membrane were normalized by dividing with the intensity of the nitrocellulose membrane signal at 1282 cm^−1^ after background subtraction. The normalisation process was also performed on the background subtracted membrane spectra. The spectrum from a single cell of intact bacterial biofilms was then recovered by subtracting the normalised nitrocellulose membrane spectrum from the normalised spectrum of bacterial cell together with membrane.

The normalized Raman spectra were then mean-centred to reposition the centroid of the data at the origin^66^. The mean-centred data were analysed by calculating the principal components (PCs), creating score plots. Each spectrum was plotted as a separate point in a multidimensional space. The characteristic peaks from the loading plots were determined using existing literature for cellular components^61^. These characteristic Raman peak assignments are associated with abundant cellular components in the substance.

### Statistical analysis

For chemical analysis, four biological replicates were obtained and for each biological replicate three technical replicates were tested. Data analysis was performed using GraphPad Prism 5 software (GraphPad Software, San Diego, California USA, www.graphpad.com). Statistical differences for a single time points were evaluated through one-way ANOVA, with a confidence level of 95% (*P* < 0.05). The statistical significance for multiple groups was determined with multiple *t* tests. Results are presented as the mean ± the standard deviation (SD), and a *P* value of < 0.05 was considered statistically significant.

For Raman spectroscopy, ten spectra were obtained from each of the three biological replicates of planktonic and biofilm samples. A commercially available software, MATLAB (version 7.10.0. Natick, Massachusetts: The MathWorks Inc., 2010) was used for processing the Raman data. A multivariate statistical method of principal component analysis (PCA) was applied to detect differences and similarities within the biofilm and planktonic spectral data sets.
